# Role of Different Types of miRNAs in Some Cardiovascular Diseases and Therapy-Based miRNA Strategies: A Mini Review

**DOI:** 10.1155/2022/2738119

**Published:** 2022-09-21

**Authors:** Safa S. Fayez, Sami Mukhlif Mishlish, Hanan M. Saied, Semaa A. Shaban, Ahmed AbdulJabbar Suleiman, Firas Hassan, Ali Z. Al-Saffar, Jameel R. Al-Obaidi

**Affiliations:** ^1^Al-Maarif University College, Medical Laboratory Techniques Department, Ramadi, Iraq; ^2^University of Anbar, College of Medicine, Department of Internal Medicine, Ramadi, Iraq; ^3^Tikrit University, College of Science, Biology Department, Tikrit, Iraq; ^4^University of Anbar, College of Science, Biotechnology Department, Science College, Ramadi, Iraq; ^5^Al-Nahrain University, College of Science, Department of Chemistry, Baghdad, Iraq; ^6^Al-Nahrain University, College of Biotechnology, Department of Molecular and Medical Biotechnology, Baghdad, Iraq; ^7^Department of Biology, Faculty of Science and Mathematics, Universiti Pendidikan Sultan Idris, 35900 Tanjong Malim, Perak, Malaysia

## Abstract

The role of microRNAs (miRNAs) in the pathogenesis of cardiovascular disease has been extensively studied. miRNAs have been highlighted as an important physiological regulator for activities like cardiac protection. miRNAs are present in the circulation, and they have been investigated as physiological markers, especially in the condition of heart failure. However, there is less compelling verification that miRNAs can outperform traditional biomarkers. However, clinical evidence is still required. In this review article, we explored the feasibility of miRNAs as diagnostic biomarkers for heart failure in a systematic study. Searching in the PubMed database to identify miRNA molecules that are differentially expressed between groups of patients with heart failure or heart disease and controls, throughout the investigation, we discovered no significant overlap in differentially expressed miRNAs. Only four miRNAs (“miR-126,” “miR-150-5p,” “hsa-miR-233,” and “miR-423-5p”) were differentially expressed. Results from our review show that there is not enough evidence to support the use of miRNAs as biomarkers in clinical settings.

## 1. Introduction

In humans, nearly 85% of the genome is to be sequenced; although, only about 2% of the genome is dedicated to coding RNA transcription. As a result, noncoding RNAs code for the huge bulk of the genome (ncRNAs). Noncoding RNAs with 200 nucleotides based on their transcript size are characterized as short ncRNAs. Long noncoding RNAs have a wide range of functions and are categorized based on regulatory element connection, chromosomal location, and structural or sequence conservation [1].

**Figure 1:**
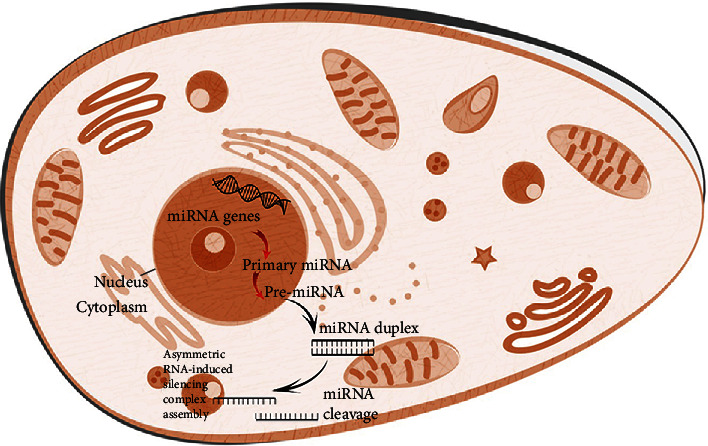
Diagram for the process of biogenesis of miRNA witin the cytoplasm. Primary miRNA synthesis begins at the nucleus.

Small ncRNA classification depends on their size or cellular location. They are 22 nucleotides long noncoding RNAs approximately, which have base pair with the target mRNA, and affect the expression of the posttranscriptional gene. RNA polymerase II primarily transcribes miRNAs as primary transcripts or prior miRNAs. Although miRNAs were identified in 1993, their significance in heart and vascular biology was not recognized until the period between 2006 and 2007 [2]. The pathogenic significance of miRNAs in the cardiovascular system was first hypothesized based on research demonstrating the abnormal miRNA expression in animal models and heart failure patients (Figure 1) [3].

MicroRNA is a type of negative regulator that can inhibit gene expression and target RNA (miRNA) and has been shown to provide a natural way to regulate gene expression [4]. The human genome has hundreds of miRNAs and thousands of target mRNAs, demonstrating the importance of miRNAs in cytopathology, cell formation, death, and proliferation [5]. miRNAs are naturally engaged in the pathophysiology of a variety of human disorders. The latest advances in miRNA technology and the detection and modification of chemical substances have opened up new ways for their applications in prediction, diagnosis, and treatment [6].

miRNA exists in almost all organisms, including nematodes, viruses, fish, plants, flies, mice, and humans and plays a role in many cellular and evolutionary functions. Researchers use miRNA microarrays to comprehensively analyze the genetic profiles of cells and tissues in different developmental or classification stages, metabolic states, and disease models to specify particular miRNA profiles [7]. miRNAs were once thought to be largely responsible for repressing target mRNA translation; however, it was recently shown that their major function in the mammalian cell is to reduce targeted mRNA levels [8]. According to recent studies, humans have about 1,000 different miRNA genes and may have as many as 20,000, with miRNA targets accounting for 20-30% of all human mRNA. As a result, most mRNAs are likely to be regulated by miRNAs to some extent. miRNAs are well regulated to operate as immunomodulators since their expression is tightly controlled. miRNA has recently been discovered to be a vital link in both the innate and acquired immune systems, and “dysregulation of miRNA” has significant worth in disease aetiology [9].

The influence of miRNAs on cardiac remodeling, which contains fibrosis and cardiac hypertrophy, has been studied in cardiac disease models. Furthermore, research shows that miRNA therapies are effective in models of myocardial infarction, ischemia-reperfusion injury, and cardiac arrhythmias [10]. In recent years, several methods for analyzing and quantifying miRNA expression were developed [11]. The quantitative polymerase chain reaction (qPCR) is a common technology that is probably one of the most accurate but most complex to utilize when the count of miRNAs reaches 300 [12]. In addition, various immunoprecipitation assay-based techniques were developed for the same purpose such as crosslinking and immunoprecipitation RNA immunoprecipitation and RNA-chromatin immunoprecipitation (RNA-ChIP) [13]. Microarrays are currently the most regularly utilized method. These technologies are extremely precise and allow for large-scale miRNA analysis [14]. The use of miRNA-targeted medicines for long-term risk reduction necessitates the evaluation of many potential negative effects [15].

### 1.1. Functions of miRNA

Analyzing and developing all miRNAs in the complex biogenesis process required multiple protein macromolecular complexes, as previously indicated. miRNA has worth in cell death, nervous system patterning, developmental timing, cell proliferation, hematopoiesis, and other aspects of proper cellular homeostasis. miRNAs are capable of altering chromatin as well as regulating the gene after the after genetic transcription phase. Moreover, aberrant levels of miRNA have been detected in numerous disorders when compared to normal counterparts thanks to the advent of genome-wide screening tolls [16, 17] (Table 1).

Considering the significance of miRNAs in regulating cell proliferation and differentiation, it is expected that miRNA dysfunction is associated with cancer. miRNA regulates cancer as an oncogene or tumor suppressor [18]. In particular, miRNAs are involved in cellular responses to oxidative stress, malnutrition, and DNA damage [19]. Chronic stress is associated with certain physical effects, for example ‘heart disease,” “immune system disease,” “inflammatory bowel disease,” and “brain dysfunction.” Because they structurally modify the structure of brain cells relevant to emotion, cognition, and memory, glucocorticoids are well-known mediators of the effects of cellular stress on neurological function and behavior [20].

### 1.2. Cardiovascular Disease Entities with the Role of miRNA as Biomarker

#### 1.2.1. Atrial Fibrillation (AF)

The role of miRNAs in gene silencing *after genetic transcription* in AF has been investigated. miRNAs have been implicated in cardiac excitability and the pathogenesis of arrhythmias. A group of microRNA inhibitors like miR-328, miR-499, miR-150, miR-409-3p, and miR-126 are discovered; nevertheless, none of the reported miRNAs has been verified. Therefore, miRNA-based AF diagnosis requires further evidence from clinical studies that study circulating miRNA [21].

#### 1.2.2. Infective Carditis

Infectious carditis, like myocarditis or pericarditis, has no obvious biomarkers. So, it is usually diagnosed through a combination of clinical trials and well-established protein biomarkers that indicate myocardial damage [22]. The ability of miRNA as a diagnostic biomarker for infectious cardiomyopathy has been studied. The increase of miR-499 and miR-208b-related cardiomyocytes in the serum of patients with viral myocarditis was reported [23, 24].

#### 1.2.3. Tako-Tsubo Cardiomyopathy

Tako-Tsubo cardiomyopathy (TTC) is a unique cardiomyopathy that causes abrupt myocardial infractions. Because there are no biomarkers to differentiate TTC from myocardial infarction, the diagnostic technique relies on morphological studies such as echocardiography and ventricular angiography (Table 2) [25].

### 1.3. miRNA Therapeutic Applications

Even though miRNAs have only recently been found in humans, a variety of miRNA-based medicines are already gaining popularity as prospective treatments [26, 27]. This is because effective, potent anti-miRNA targeting strategies were swiftly discovered and tested in vivo in preclinical animals [28]. miRNAs can be efficiently suppressed using single-stranded antisense oligonucleotides, similar to those used to silence mRNA gene transcripts using small interfering RNA [29, 30]. To stabilize these anti-miRNA molecules, reduce the effective dose for *in vivo* administration, and boost their toxicity and tissue absorption, various chemical alterations have been made [31].

Although some treatment structures and medications are employed to treat and palliate care difficulties, they are usually used to delay the onset of the disease or obtain reliable treatment, especially in people with a family history of hereditary coronary artery disease [32]. More powerful oligonucleotides, especially interfering RNA, are effective cellular targets in signaling pathways, and delivery systems with next-generation drug platforms are all examples of how genetic engineering has evolved in this field [33]. miRNAs may be attractive therapeutic targets because they are involved in the pathophysiology of the aforementioned cardiovascular risk factors [34, 35]. Many potential side effects must be considered when using miRNA-targeted therapies to reduce long-term risks.

Due to the biodiversity of miRNAs, repair treatment requires targeted application of inhibitory and mimetic miRNA chemicals and favorable pharmacokinetic characteristics [36, 37]. Acute miR-15 antagonism may help the heart repair and function by promoting the proliferation of cardiomyocytes after myocardial infarction [38]. However, long-term anti-miR-15 treatment can trigger whole-cell proliferation and promote tumor formation [39]. Anti-miR-15 targeted drugs and fast-metabolizing drugs that temporarily reduce miR-15 after myocardial infarction can avoid these adverse systemic reactions [40]. Anti-miR122 (miraversen) has strong safety and effectiveness characteristics. According to phase II results, more than half of patients receiving anti-miR122 treatment have undetectable hepatitis C virus levels after treatment. Although anti-miR122 appears to have a lot of potential in the treatment of hepatitis C, its usage in the treatment of hyperlipidemia requires more research [41].

## 2. Materials and Methods

### 2.1. Search Strategy

**Figure 2:**
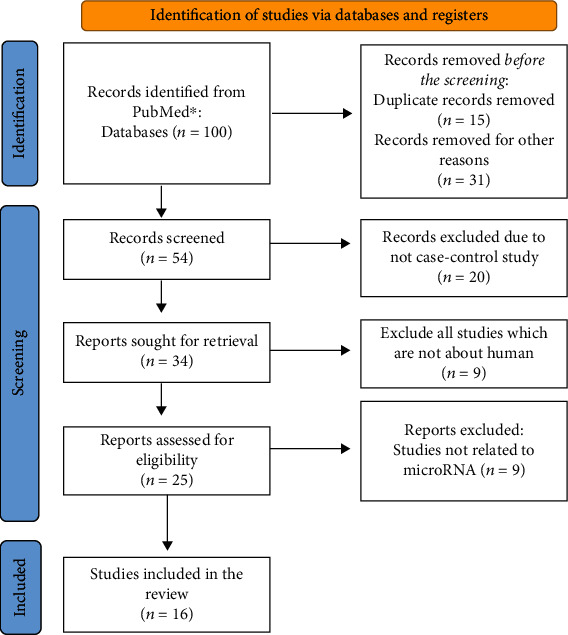
Search strategy for the miRNA expression associated with heart failure based on the PubMed database.

The search of the PubMed database was done using a best-match algorithm to retrieve all published evidence for the association between miRNA expression and heart failure. Keywords used in the search strategy were miRNA, microRNA, and miR as biomarkers for the diagnosis of heart failure. We searched for articles published in 2021 (Figure 2).

### 2.2. Incorporation and Elimination Criteria

Articles were involved that are providing independent source data and evaluated miRNAs as biomarkers for heart failure diagnostic in humans. Therefore, in research articles, reviews, meta-analyses, case reports, abstracts, correspondence or reviews, and method studies, only articles published in English were considered. Studies without miRNAs analyses, not including cohort or control data, focused on other diseases, or were used for other purposes were also excluded. Articles were also excluded if they did not specify miRNA expression, did not focus on the heart, or did not compare the miRNA expression in cardiovascular patients with controls after full-text evaluation.

### 2.3. Qualitative Assessment

Authors extracted data independently from each study that was eligible and abstracted the following information: the number of control and diseased, source of RNA extraction, sample extraction method, conclusions, references of study, and those studies which found miRNA related to cardiovascular diseases.

## 3. Results

### 3.1. Search Results

All articles were identified from various sources defined in Methodology. A total of 54 articles were identified using PubMed's online database. Thirty-four abstract reviews were excluded because the full text did not meet the inclusion criteria. Twenty-five human miRNA studies were used as diagnostic biomarkers for heart failure. Results from 16 clinical studies were selected because they compared the expression of miRNA between patients and healthy controls.

### 3.2. Study Characteristics

In Table 3, the characteristics of 16 studies that evaluated more than 100 miRNA prognostic biomarkers included in the systematic review were described. Studies have been conducted in several cities or countries. Studies included a total of 3,350 patients, and analyses for different cohort sizes were considered in individual studies. The miRNA expression was found to be analyzed in fresh/preserved tissue, serum, and plasma samples. All studies investigated the miRNA expression using RT-PCR. Two studies did not provide relevant or found miRNAs. There were no control cases in one study, and three studies did not reveal the origin of the samples.

## 4. Discussion

Fourteen eligible studies regarding the inclusion criteria have coincided. Common miRNA expression profiles across multiple domains of the disease and specific miRNA expression profiles for each domain were identified. This research will add to our knowledge of the miRNA-related regulatory processes in heart failure and aid in the identification of diagnostic biomarkers or treatment targets for patients with heart diseases. Through a systematic study, we observed over 100 differently expressed miRNAs between groups of patients with heart diseases and controls. We identified more than 100 miRNAs with different expression levels between groups of patients with heart failure and controls. In three studies, new biomarkers for heart failure have been investigated. One study concluded that the connection of miR-150-5p with disease severity, maladaptive remodeling, and outcome supports a pathophysiological association of advanced heart failure with downregulated miR-150-5p expression [44].

Studies have shown that different miRNA combinations are valuable biomarkers for heart failure and for differentiating heart failure reduced ejection fraction (HFrEF) from heart failure preserved ejection fraction (HFrEF) [53, 56, 57]. The inclusion of natriuretic peptides in certain biomarker combinations could change their utility. miRNA biomarkers may aid diagnostic efforts for a subset of heart failure patients [47].

Studies have shown that miR-3908, miR-3135b, and miR-5571-5p may serve as novel biomarkers for heart failure, and the levels of these miRNAs can be used to determine risk assessment [53]. On the other hand, a study found that patients with cardiac sarcoidosis-related heart failure had changed the expression of miR-223 and miR-126 in their peripheral blood. The findings of this research show that circulating miR-126 and miR-223 could be used as next-generation biomarkers for sarcoidosis diagnosis in patients with heart failure [45]. Finally, common miRNA expression profiles in diverse areas of heart failure disease were found, as well as distinct miRNA expression profiles in each area. This shows that some miRNAs may have a role in common heart disease pathways. A summary of this study could aid in the selection of potentially helpful biomarkers for clinical practice and encourage further research into heart failure treatment targets.

## 5. Conclusion

Breakthroughs in understanding cardiac pathophysiology are urgently needed, which can subsequently be applied to the clinical environment through the development of innovative diagnostics and therapies. miRNAs influence the progression and development of ventricular failure and hypertrophy, making them key regulators of cardiac responses to pathological stresses. The use of miRNAs to improve heart regeneration while also boosting cardiac contractility and lowering fibrosis opens up new therapeutic possibilities. Clinically, miRNAs can be valuable in the diagnosis and monitoring of markers. Our assessment of the usability of miRNAs as diagnostic markers for heart diseases reveals that miRNAs are not well supported in the current clinical situation. To determine the certain value of miRNAs as diagnostic indicators, future experimental investigations utilizing the same approach must be devised and performed on large sample numbers.

## Figures and Tables

**Table 1:** List of miRNA functions related directly or indirectly to cardiovascular diseases and therapy.

Cellular function
Cell fate specification
DNA repair
DNA methylation
Cell proliferation
Cell differentiation
Developmental timing
Cell cycle control
Angiogenesis control
Pattern formation
Morphogenesis regulation
Synapse function fat metabolism
Stem cell maintenance
Insulin secretion
Resistance to viral infection
Inflammation
Proinflammatory stimuli
Apoptosis
Immunomodulation
Anti-inflammatory stimuli
Neuronal differentiation
Neurogenesis
Neuroprotection

**Table 2:** The miRNA ability to be used as diagnostic biomarkers for miRNA in-stent restenosis, arterial fibrillation, infective carditis, and Tako-Tsubo cardiomyopathy.

miRNA in-stentrestnosis	Infective carditis	Artrial fibrillation	Tako-Tsubo cardiomyopathy
miR-21	miR-208b	miR-499	miR-1
miR-143	miR-499	miR-328	miR-16
miR-145		miR-150	miR-26a
miR-100		miR-409-3p	miR-133a
miR-21		miR-432	
miR-145		miR-126	

**Table 3:** Study characteristics of eligible researchers conduct the association between heart failure and miRNA expression.

Number of subjects		Source of RNA	Conclusions	Found correlated miRNAs	Origin	Platform of RNA
Control or healthy	Patients					
10	10	Plasma	Findings suggest that ceRNA networks may play a key role in the development of heart failure and may have immune response functions hsa-miR-8485, hsa-miR-26b-5p, TUG1, hsa-miR-940, GAS5, and HOTAIR which were identified as key genes [42].	hsa-miR-26b-5p hsa-miR-8485 hsa-miR-940		RT-qPCR
30	28	Plasma	exo-miR-92b-5p can be a biomarker candidate for diagnostic [43].	exo-miR-92b-5p	Ningbo (China)	RT-qPCR
5	10	Serum	Findings suggest that miR-150-5p could be a new circulating biomarker for progressive heart failure [44].	miR-150-5p		RT-qPCR
9	18	Peripheral blood	Development of next-generation biomarkers miR-126 and miR-223 for cardiac sarcoidosis diagnosis in patients with heart failure [45].	miR-126 and miR-223	New York (USA)	RT-qPCR
Screening study 14 + validation study 15	47+135 with other heart problems	Plasma	Seven miRNAs were identified to distinguish between heart failure and non-HF causes of dyspnea [46].	miR-103 miR-142-3p miR-30b miR-342-3p		RT-qPCR
75	75 of HFpEF and 75 of HFrEF	Plasma	Various miRNA combinations can be used as HF biomarkers. The addition of natriuretic peptides to certain biomarker combinations can change their value. In subpopulations of patients with HF, miRNA biomarkers may aid diagnostic techniques [47].	miR-30c miR−146a miR−221 miR−328 miR−375	Dublin (Irlend)	RT-qPCR
41	90 with different heart diseases	Plasma	The increasing acuity of heart failure was linked to decreasing levels of circulating miRNAs [48].	miR-18b-5p miR-223-3p miR-301a-3p miR-423-5p miR-652-3p	Denmark	RT-qPCR
39	45 DCM patients	Serum	Findings conclude that miR-423-5p levels in the blood could be useful as a biomarker for diagnosing dilated cardiomyopathy-related heart failure [49].	miR-423-5p miR-126 miR-361-5p miR-155 miR-146a	New York (USA)	RT-qPCR
55 of ICM and 51 of NICM		Plasma	The miRNAs miR-126 and miR-508-5p could be useful in the diagnosis of chronic heart failure patients [50].	miR-126 miR-508-5p miR-34a miR-210 miR-490-3p miR-513-5p miR-517c miR-518e miR-589 miR-220c miR-200a miR-186 miR-7i miR-200b miR-595 miR-662	Hangzhou (China)	RT-qPCR
Cohort I (Barcelona) comprised 834 chronic heart failure patients. Cohort II (Detroit) comprised 1369 chronic heart failure patients		Plasma	This study found an association between “miR-1254 and miR-1306-5p” and the risk of hospitalization and death from heart failure in two independent cohorts of patients with heart failure. [51].	miR-1254 miR-1306-5p	Barcelona (Espain), Detroit (USA)	RT-qPCR
32	42	Plasma	miR-150-5p is a predictor of overt heart failure that is independent of other factors, and it could be utilized to screen patients for this condition [52].	50 miRNAs found	Germany	RT-qPCR
3	13	Serum	The findings of this study conclude that miRNAs (miR-3135b, miR-3908, and miR-5571-5p) can be used as biomarkers for heart failure and to distinguish HFrEF from HFpEF [53].	miR-3135b miR-3908 miR-5571-5p	Beijing (China)	RT-qPCR
80	80	Plasma	The plasma biomarkers “circulating long non-coding RNA” taurine upregulated gene 1 and NT-proBNP were found to be beneficial in the diagnosis of HFpEF in hypertensive people [54].			RT-qPCR
21	68	Plasma	The alpha/beta hydrolase fold domain 4 mRNA biomarkers could diagnose acute coronary syndrome early and stratify severity, thereby improving health outcomes [55].		Egypt	RT-qPCR

HFrEF: heart failure reduced ejection fraction; HFpEF: heart failure with preserved ejection fraction.

## Data Availability

Data is available.
